# Health Privacy Information Self-Disclosure in Online Health Community

**DOI:** 10.3389/fpubh.2020.602792

**Published:** 2021-02-04

**Authors:** Wang Yuchao, Zhou Ying, Zangyi Liao

**Affiliations:** ^1^School of Economics and Business Administration, Hubei University of Arts and Science, Xiangyang, China; ^2^Economics and Management School, Wuhan University, Wuhan, China; ^3^School of Political Science and Public Administration, China University of Political Science and Law, Beijing, China

**Keywords:** online health community, health privacy information, privacy calculus, self-disclosure, empirical study

## Abstract

The scarcity of medical resources is a fundamental problem worldwide; the development of information technology and the Internet has given birth to online health care, which has alleviated the above problem. The survival and sustainable development of the online health community requires users to continuously disclose their health and privacy. Therefore, it is a great practical significance to find out the factors and mechanisms that promote users' self-disclosure in the online health community. From the perspective of individual and situation interaction, this study constructed influencing factors model of health privacy information self-disclosure. Finally, we collected 264 valid samples from the online health community through online and offline questionnaire surveys and then use the SPSS20.0 and AMOS21.0 to conduct exploratory factor analysis, confirmatory factor analysis, scale reliability and validity analysis, and structural equation model analysis. The main findings are as follows: trust in websites and trust in doctors reduce the privacy concern. The privacy trade-off will not occur when trust is enough to offset the privacy concerns caused by personalized services, reciprocity norms, and other factors. Second, reciprocity norms are inevitably compulsive, which will increase privacy concerns. However, based on voluntariness, reciprocity norms can enhance user trust. Third, service quality caused by personalized services not only enhance the social rewards of users but also eliminate the privacy concern. Fourth, users' health privacy attention and information sensitivity are too high to decrease the influence of user' privacy concerns on personal health privacy information disclosure. The conclusions of this paper will help us to supplement privacy calculus theory and the application scope of the attention-based view. The proposed strategy of this article can be used to stimulate the information contribution behavior of users and improve the medical service capabilities in online health community.

## Introduction

The shortage of medical resources is a fundamental problem worldwide. Fortunately, the development of information technology and the Internet has generated Online Health Care, which makes up for this shortage. Especially, the online health community service enables more patients to obtain effective information to improve their own situation. According to the research report released by PEW Research Center, 75% of American patients search for information about personal health and medical treatment on the Internet (1), including physical conditions, symptoms, and advice on treatment options. This new type of online medical model is quickly received by people worldwide (2, 3) because of cost saving, privacy protecting, embarrassment reducing, higher search efficiency, and individual matching. The number of online health communities has grown rapidly all over the world in the past few years. According to authoritative data released by Startup Health, the US digital medical entrepreneur accelerator, the global Internet medical investment and financing amounted to 3.9 billion dollars in the first half of 2019, a year-on-year growth of 34.5%, and Chinese Internet medical users alone reached 195 million.

The whopping business potential of online health community attracts scholars and practitioners to consider how to achieve its sustainable development. As online health care relies heavily on the information contributions of users, online health care community, a special type of online community based on Web2.0 technology, requires continuous content contribution of users for its survival and sustainable development (4). This contribution behavior was named network self-disclosure (5). User-contributed content refers to the communication between users and other members in online communities (6). Users focus on who contributed the health information and the content in the online health community is directly related to self-disclosure of illness. Therefore, understanding the factors that influence self-disclosure behaviors of personal health information has great theoretical and practical significance.

Self-disclosure may be caused by different factors in different situations, and in previous health information disclosure researches have focused on privacy issues caused by different situations, such as electronic commerce (7, 8), electronic health (9, 10), social networking sites (11, 12), digital media (13, 14), location aware market (15), etc. Many scholars believe that it is reasonable to analyze user information self-disclosure based on privacy calculus theory (5, 16, 17). Privacy calculus refers that users compare potential risks and expected benefits form a subjective assessment of costs and benefits and decide whether to disclose personal information before disclosing personal information (16, 17). These studies suggest that perceived benefits have a positive impact on self-disclosure intention, while perceived risks have a negative impact on it. Originated from equity theory and justice theory, this model attempts to solve the calculation of the income and expenditures when users face with privacy disclosure. Its purpose is to achieve the same gains with less effort or to obtain higher benefits with the same effort (6). Although few researchers have focused on online health community (1–3), there are still some research points needed to be added.

First, privacy calculus theory points out that the trade-off between perceived risks and perceived benefits is the prerequisite for users' disclosure intention. Studies based on this theory ignore the role of trust. Wang and Lin found that users' trust negatively affects perceived risks (15, 17). The trade-off will disappear when the users' trust is enough to eliminate the perceived risks. In fact, exploring this problem can comprehensively reveal the generation mechanism of users' self-disclosure intention. Therefore, this study aims at examining whether trust can influence the privacy trade-off. Moreover, based on the context of Internet knowledge sharing community, most of the previous studies focused on the knowledge sharing behavior of users in the Internet that will be actively promoted by reciprocal norms (18, 19). However, the reciprocal norms based on the principle of social exchange in the online health community are compulsive, and they may also have a negative impact on users. Voluntary-based reciprocity norms make users form grateful trust in return, while the reciprocity norms based on forced exchange may increase the perceived risks of users (19, 20). Therefore, the second purpose of this study is to find out whether the impact of reciprocity norms changed when the situation shifts from an Internet knowledge sharing community to an online health community.

Second, previous studies based on location-aware market have confirmed that personalized services have a positive impact on perceived benefits and perceived risks (15). On the one hand, personalized service will bring a lot of convenience to network users; on the other hand, it will cause some health privacy concerns that are not conducive to the promotion of personalized services. Therefore, it is urgent to eliminate the negative effects of personalized services (21). Previous studies have shown that service quality is negatively correlated with privacy concerns (22), whereas scarce studies have confirmed that service quality on the network medical platform can reduce the privacy concerns brought by personalized services. It is the third problem that this study tries to solve.

Third, from the perspective of interaction between individual and environment, privacy calculus is not only affected by virtual health community services but also affected by individual and information factors. Another potential problem is what leads to the perceived benefits and perceived risks of health privacy information sharing. Obviously, the degree and scope of users' health privacy attention will inevitably affect their attitude toward personal health information sharing on health websites. The more users pay more attention to personal health privacy, the more they worry about the potential risks of health information exchange and the lower users' perception of gaining benefits from the website. In addition, a more important reason lies in the health privacy information itself. Once the sensitive health information has been leaked, users will have a strong sense of shame and psychological pressure, so they have high privacy concerns about this kind of information, and sharing sensitive health privacy will also be affected when users perceived low benefits. However, few previous literatures have researched the relationship between privacy concerns, information sensitivity, and privacy calculus. This is the fourth question of this study, that is, whether privacy concerns and information sensitivity are the source of perceived benefits and privacy concerns and how do they affect self-disclosure.

Based on privacy calculus model (23) and theory of interaction between individual and environment (24), this study aims to solve three theoretical problems that have not been considered in the previous literature. First, we propose that the reciprocal norm established on the online medical platform is compulsive, which will increase the perceive risks by users. Privacy calculus model points out that users decide whether to disclose personal health information based on the trade-off between perceived benefits and perceived risks. Reciprocal norms based on voluntary conditions can enhance users' trust. Then, will the privacy trade-off happen when trust is sufficient? Second, according to the previous researches, the personalized service can not only enhance their perceived benefits but also cause their perceived risks of privacy disclosure. We assume that the service quality can enhance the perceived benefits of users and better eliminate the perceived risks of personalized services. Third, since few researchers studied the relationship between health privacy attention, information sensitivity, and privacy calculus, we attempt to verify whether privacy concerns and information sensitivity are the root causes of perceived benefits and privacy concerns and how do they affect self-disclosure.

## Theoretical Background

### Self-Disclosure

Online health community is a virtual community where a group of people communicate with each other about health and medical care so as to find information, help, emotional support, and communication opportunities or to influence public. The online health community has various functions: connecting people with the same experience without the limits of geographical and social status, maintaining community engagement without focusing on time, location, and planning changes, and improving health outcomes and quality of life. People participate in the online health community not only to acquire professional medical science knowledge but also to acquire experience and knowledge from the experience of community members (25). The online health community allows individuals to voluntarily or involuntarily disclose personal information to others. This is considered a form of self-disclosure technology (26). With the rapid development of the “Internet+” industry, the online medical service platform has sprung up. As an example of Internet medical service, the online health community started early and has had a huge impact on the doctor–patient behavior in the past decade. The voluntary health information sharing behavior of patients can not only enrich the medical information content of virtual health community and help other patients with similar diseases get reasonable treatment but also help doctors check the effect of diagnosis and treatment, alleviating the predicament of medical resources shortage caused by health information asymmetry. Compared with the encouragement of users to disclose information on traditional social networking sites, encouraging users to disclose personal health information requires more trade-off because patients' health information is more sensitive than other information (27).

### Privacy Trade-Off

The core concept closely related to health information self-disclosure in the existing literature is privacy (6, 15, 28). In these studies, Bé Langer and Crossler regarded privacy as the ability of a person, an online medical platform user, to control his/her health privacy information (29). This definition is widely accepted. Accordingly, privacy risks may be related to the uncertainty factors caused by misuse of privacy information and will bring losses to users (30). To avoid potential losses of privacy risks, users tend to protect their health privacy. However, the professional medical knowledge is in the hands of doctors on the website and is difficult to obtain from other channels. Users must give up their privacy protection rights and provide personal information to the website in exchange for online medical services. The conflict leads to privacy trade-off of users. Dinev et al. believe that users will disclose personal health information when the benefits obtained from personal privacy disclosure can compensate for the loss caused by personal health information disclosure (30). This privacy calculus theory has been widely adopted in various situations, and it better explains the health privacy trade-off (5, 15–17).

Previous behavioral research have shown that individual behavior intention is the result of the interaction between individual factors and situational factors. Privacy trade-off is the prerequisite of individual behavior intention, and it will be influenced by both online community factors and user-related factors (31). In the past, the research on privacy calculus has not applied the human situational interaction theory to study the privacy trade-off. On the contrary, the human situational interaction theory has been widely used to analyze the psychological process before the formation of individual attitudes and behaviors (31, 32). Furthermore, scholars believe that individual behavior is rooted in their cultural background and closely related to the specific personality. We need to study privacy from the interactive effects of individuals and cultural background to meet the situational facts (33, 34). Therefore, it is necessary to explore the applicability and influencing factors of privacy trade-off in the medical context from the perspective of interaction between personality and culture background.

### Privacy Calculus Theory

In previous studies on user privacy information disclosure, scholars mainly focused on two scenarios: e-commerce and self-disclosure technology environment. In the study of e-commerce, researchers mainly focus on the analysis of antecedent variables that affect the willingness of self-disclosure. We explore the impact of service provider characteristics and user interpersonal differences on privacy trade-off to expand the privacy calculus model (35). In the study of self-disclosure technology environment, it mainly includes social network, timely communication, microblog, blog, mobile application, etc. (6, 17). Studies have shown that privacy risk was affected by perceived severity, perceived control, personality service, and trust (15, 17, 35). Although the research on privacy risk has received extensive attention from scholars, more empirical evidence is needed to do research on the perceived risks of online medical platform users. In addition, previous studies have pointed out that perceived benefits are affected by entertainment revenue, practical revenue, personalized service, self-presentation, and other factors (6, 17), while the perceived benefits of social rewards and practical rewards are rarely involved. Research on perceived revenue based on online healthcare is even rarer. This requires us to supplement the theory of this issue.

Privacy calculus model provides a good theoretical framework to analyze the behavior of privacy information disclosure, but there are still some research points to be filled. First, from the perspective of service, personalized service and service quality complement each other and jointly affect the perceived benefits of users. For personalized services, a previous study based on the location-aware market has confirmed that personalized services have a positive impact on perceived benefits and perceived risks (15). Personalized services will bring convenience and better network experience to network users, but a large amount of information may touch personal privacy issues, which will greatly enhance users' perceived risks and reduce users' recognition of personalized services. Therefore, it is very urgent to eliminate the negative effects of personalized services (21). Previous studies have shown that service quality is negatively correlated with perceived risks (22). However, few studies have examined whether the service quality improvement on the network medical platform can reduce the risk of personalized service. Second, the privacy calculus model points out that the trade-off between perceived risks and perceived benefits is the prerequisite of users' intention (23, 30). However, these studies ignore the role of trust. Wang and Lin found that users' trust negatively affects perceived risks (35). Will this trade-off disappear when trust is sufficient to eliminate perceived risk? Third, in fact, the reciprocity norms are also the operation foundation of virtual communities. Unrequited behavior is short lived and cannot ensure the stability of community members (36). In addition, some scholars have studied knowledge sharing in online communities from the perspective of social capital. They believe that reciprocity norms represent a perception of fair knowledge exchange among community members. The results show that reciprocal norms have a significant impact on knowledge sharing (37, 38). Online community knowledge holders are based on voluntary reciprocity norms, while medical information resources are scarce and difficult to obtain. Such reciprocal norms in special community situations are compulsive. How does it affect the results of health privacy self-disclosure is also worthy to further study.

## Research Model and Hypotheses

**Figure 1:**
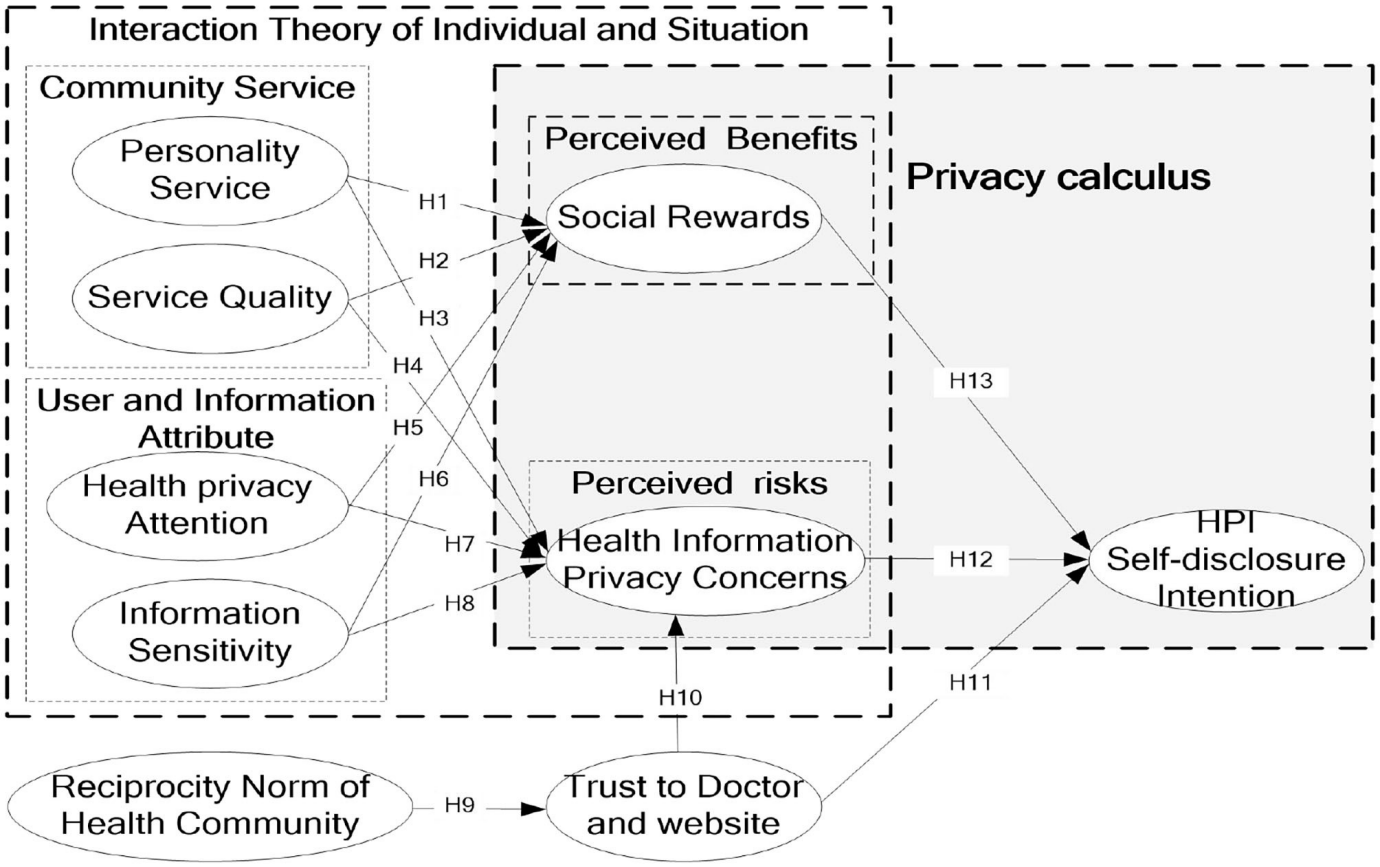
Research model.

Figure 1 shows the research model based on the above. This study proposed and tested several hypotheses to understand the relationships among several constructs.

### Impact of Community Service on Perceived Benefits

Social rewards refer to the satisfaction, pleasure, and happiness in the use of online health community. Personalized service is defined as the ability to customize content and service for individuals based on their personal preferences, knowledge, and behavior. Users may be motivated to disclose personal information in exchange for personalized services or access to health information. When users use the network medical community, the customized service of the users provided by the medical website will fit the users' usage habits, values, and specific medical needs. After these personalized needs are met, the convenience of network medical service perceived by users is stronger, and the users' satisfaction of website is higher. At the same time, users is also dependent on medical and health website, immersing themselves in the happiness of using medical websites. The sense of satisfaction and pleasure was generated by medical service using, which is the core of social rewards. Based on above discussion, this study proposes the following hypothesis:

H1: Personality service has a positive impact on social rewards.

Network service quality refers to the overall evaluation of users on the excellent degree of service that was provided by the network service provider. When seeking medical advice in the Internet health community, website users evaluate the quality of medical website services only based on the level, which they perceive as valuable during interaction with the website, such as the professionalism of the web design, the convenience of use, the stability of the system, and the reliability of privacy protection. These factors directly improve users' positive sensory emotion and, using experience during the use of the webpage, create the sense of satisfaction and pleasure for users. Thus, the more sophisticated service was provided by the online health community, the richer the content searched by users on the website, the stronger the users' satisfaction. This sense of gain and joy is the most important part of social rewards thus, this study propose the following hypothesis.

H2: Service quality has a positive impact on social rewards.

### Impact of Community Services on Privacy Concerns

Research has proved that collecting a large amount of personal information to perform personalized services will cause heavy privacy risks. For example, the identity information of users will reveal the real identity of users in the daily society, which may cause potential key privacy issues. Human search events of the users will occur if the users' identity information and other personal health information are processed unreasonably. The mined information of users may be used for fraud, network attack, or illegal trading of user information for commercial purposes. In the network medical community, the users' browsing history will enhance their privacy concerns because it records the users' preferences and the real demand trajectory. Although personalized information and services provide users with more added values during their network usage, users still pay attention to how their information was collected and used to perform the personalized service. Therefore, this study proposes the following assumption:

H3: Personality services have a positive impact on privacy concerns.

Privacy concern refers to, under the circumstance of online health community, an individual's concern about his or her own personal information, including the transference and exchange of such information. Online service evaluation is slightly different from the traditional service evaluation. Internet users pay less attention to every detail of web services during web browsing but pay more attention to the overall process and the final results of services. Then, they evaluate the services as a whole. If there is no stimulation to arouse users' privacy concerns, users' evaluation of network service quality is limited to the network service itself and does not involve other aspects. The network medical service platform put itself into consideration for users, including considering their needs and paying attention to their interests. All these will help users reduce privacy concerns. Sweetey, Soutar, and Johnso pointed out that perceived financial risk and perceived performance risks have a significant negative impact on service quality (functional quality and technical quality). High-quality services can shorten the relation distance between users and medical websites, enhance emotional commitment, and reduce users' privacy concerns. Therefore, this study assumes the following:

H4: Service quality has a negative impact on privacy concerns.

### The Impact of User and Information Attribute on Social Rewards

Faced with the same health privacy information, individuals will show different information sensitivity. In the same context of privacy policy, perception of privacy disclosure is not greatly affected by the type of information when users perceive less sensitive information that they need to provide. Under these circumstances, the information boundary is open, and users are willing to provide personal health privacy information. Therefore, users show positive experience to the website. Nevertheless, users pay more attention to the privacy policy of the online medical service platform and show stronger recognition ability and lower trust degree when they perceive higher sensitive information that they need to provide. In this case, users feel that their information control abilities were weakened and believe that more serious potential consequences may occur. Thus, users' medical service community experience proves to be negative (39). From the above discussion, we can point out that social rewards are exactly a kind of user experience in the online medical service community. Therefore, this study proposes the following assumption:

H6: Information sensitivity has a negative impact on social rewards.

From the perspective of development process, the definition of health privacy attention remains controversial. No unified definition has been formed so far. According to the research of Ocasio and Nakarni et al., this study defines health privacy attention as the capability of noticing, encoding, explanting and focusing about the personal health privacy information caused by users' provision of personal health privacy information to the website whether they are informed or not. Previous studies have shown that users' health privacy awareness varies with the health privacy information processing capability (40). This kind of difference will cause the difference in users' perceived value in the online medical service community. For instance, the sense of entertainment, social relationship capital, emotional support, and other social benefits will also vary with privacy awareness. Usually, people will pay more attention to the current short-term benefits and are too optimistic about the benefits brought by social media due to the low privacy awareness and the uncertainty of the future. The current social benefits of users will increase when people underestimate the future impact of a privacy disclosure (41). However, when the health privacy attention is high, users pessimistically believe that the health privacy disclosure may happen in the medical community at any time. This will cause great pressure on users' psychology, and obviously, the social rewards will be reduced. Therefore, this study proposes the following assumption:

H5: Health privacy attention has a negative impact on social rewards.

### The Impact of User and Information Attribute on Privacy Concerns

Phelps et al. studied different problems and categorized information twice. The first category consists of four layers: demographic information, financial information, life style, and personal experience information. When he researched about shopping problems, he came up with the second classification of information: demographic and statistical information and shopping experience, lifestyle, and financial information. As the sensitivity of information increases, users' privacy concerns will gradually increase. For example, when users are required to provide their own financial information, their privacy concerns are much higher than those caused by demographic information. Malhotra et al. also believe that sensitive information has a greater impact on privacy concerns, while less-sensitive information has a litter impact on privacy concerns. The sensitivity of information determines the importance of information because the more sensitive the information is, the more serious the negative impact of disclosure on the individual will be. Users are psychologically afraid of the disclosure or abuse of sensitive information, so this study proposes the following assumption:

H8: Information sensitivity has a positive impact on privacy concerns.

Health privacy attention is a kind of health privacy information processing capability associated with health privacy disclosure. It is an individual subjective feeling in a specific privacy situation. It reflects the users' internal concerns about the potential exposure of health privacy information (42). It mainly focuses on how information can be obtained by others and how information can be used by others. Personal health privacy information is closely watched by users. The more sensitive information users pay attention to, the more privacy leakage they will find. Once the incident of privacy information leakage occurs, it will cause huge and direct losses to users. These possible situations will objectively arouse users' concerns about privacy leakage, and such concerns are called privacy concerns. Based on the above discussion, this study proposes the following assumption:

H7: Health privacy attention has a positive effect on privacy concerns.

### The Impact of Reciprocal Norm on Trust

Trust refers to the confidence of one party in the transaction that the other will fulfill its obligations to the other. According to previous research, trust has been divided into two dimensions, namely, trust in the website service provider and trust in the website service personnel. (In this study, trust can be divided into two dimensions: trust in online medical websites and trust in online doctors.) For example, users believe that online doctors will comply with usage rules of social networks and will not embezzle or sell patients' health privacy information. The trust in the service provider means that users believe that the service provider of the network medical platform is appropriate, reliable, and ethical.

Reciprocal norm was based on the theory of social exchange and was first put forward in the field of knowledge sharing. The knowledge owner is willing to share knowledge only when he/she can predict the direct or indirect equivalent return or reciprocal benefit from the knowledge receiver, such as the help, care, and love of the receiver to the sharer. This is reciprocal norm. In this study, the reciprocal norm refers to the interactive rule established by the website operators among the website doctors, users, and backstage staff based on the equal exchanges. This rule is conducive to form a benign social interactive environment in the virtual health community. In this environment, website operators, doctors, and patients can benefit from the interaction.

Reciprocity norm is a common social norm that requires people to repay those who have helped you and expressed kindness to you. Individual perception of reciprocity will produce positive and negative feelings—gratitude and guilt. In the interaction process among users, doctors, and network operators in the virtual health community, users will form a positive emotion gratitude based on reciprocity when users realize that doctors and online medical platforms have provided help or valuable treatment information. Jones and George's research shows that emotion can affect judgment, and people usually use emotion as the basis of judgment when deciding whether to trust each other. Gratitude is a kind of positive emotion of relationship maintenance. Gratitude based on reciprocity norms plays an important role in enhancing trust in relationships. Therefore, this kind of reciprocal norm was perceived by users of the online medical platform that can encourage users to return goodwill to medical websites and website doctors so as to strengthen mutual trust in the relationship between the two parties.

H9: Reciprocal norm has a positive effect on trust.

### Impact of Trust on Privacy Concerns

In management practice, Rotter defines trust as a mutual expectation based on the existing relationship. This expectation requires both parties to abide by the oral or written commitments that have been reached. In this research, trust refers to users' belief that online medical websites will take measures to protect their health privacy information and ensure such health information will not be monopolized or abused. In the field of online health care, trust helps users to make disclosure decisions under asymmetric information and promotes the exchange of health information. For example, when users and the website builder have established a foundation of trust, they tend to follow established procedures to do information exchange during communication. The established procedures have been set up by merchants to protect the privacy information of users, and they are the default commitments between the merchants and the users based on the pre-existing trust. This default commitment will reduce the users' privacy risk perception, and once the privacy risk disappears, the privacy concern will decrease naturally. Therefore, based on the above, this study proposes the following assumption:

H10: Trust has a negative impact on privacy concerns.

### Influencing Factors of Self-Disclosure Intention

The integrity of information shared by people on the network can judge the degree of trust. When users trust other users in the medical community or the community itself, they will share more true health information online, instead of concealing their health information from the network because of distrust to protect health privacy. In addition, the research of Derlega and Chaikin suggests that the anonymity of the network helps more users share their privacy with other users who do not know them because they do not know their real identity from one another. Obviously, this behavior does not come from the trust in other users but from no privacy leakage owing to the specific secrecy of this social circle. Even if the users' health information was leaked, it will not bring any loss to users' reputation or property because the information cannot have a corresponding relationship with each user.

Network anonymity is a special reciprocity norm, the trust between the internet health community and each user is established based on this reciprocity norm. Users believe that the website will not publish their real identity information, and the website believes that users will actively participate in the construction of the website. With this default rule as a guarantee, users will disclose personal health privacy information on medical websites. Based on the above, this study proposes the following assumption:

H11: Trust has a positive effect on self-disclosure intention.

The greater the privacy risk perceived by social network users, the less likely they are to disclose personal information. For example, in a survey of 369 Internet users, Tamara Dinev et al. found that perceived Internet privacy concerns negatively affect the personal information disclosure intention. In online health community, users realize that the disclosure of health information may cause personal privacy leakage, especially some highly sensitive and infectious disease information. Once such information spreads on the Internet, it will cause interpersonal discrimination. For example, if other network users' human flesh search the personal patient history and publish such information in online media, it will directly cause reputation damage to the users in the virtual health community. On the other hand, the health information, such as disease type, telephone number, and address, exposed by individuals on the virtual health website creates opportunities for network service providers to obtain illegal profits by peddling and selling user information and provide an information basis for lawbreakers to use this information for medical fraud, which may pose a threat to the users' life safety. The privacy concerns make users more inclined to protect disease information and personal information, especially some highly sensitive disease information. Based on this, this study proposes the following assumption:

H12: Privacy concerns have a negative effect on self-disclosure intention.

Individuals gain pleasure and happiness through interaction with friends; meanwhile, this pleasure helps them maintain and develop friendships. Self-disclosure helps them to develop and maintain social relations. For example, it is essential to show common interests and hobbies when making new friends. Online medical websites users disclose their hobbies to find someone with common interests. Individual participation in reciprocal social interaction is closely related to the fair exchange of information. Users provide their personal information to others, and at the same time, they expect information from others of the same amount and depth in the online health community. Therefore, users will self-disclose if the social rewards has been adequately compensated. Based on the above, this study proposes the following assumption:

H13: Social rewards have a positive effect on self-disclosure intention.

## Research Methodology

### Participants and Procedures

We sent out both online and paper-based questionnaires. For the online questionnaire, we write the questionnaire items into the questionnaire star and send out the questionnaire through WeChat. To ensure an adequate response rate in this study, the Quality Inspection Engineer of Wuhan Toby Technology Company Limited helped us send survey questionnaire network link in China. This Quality Inspection Engineer frequently collaborates with other enterprises staff on product quality optimization. He contacted the staff of different companies through an online invitation to participate in our questionnaire survey. Moreover, for the paper-based questionnaire, students in business management at Wuhan University delivered coded invitation packets to each participant. Each packet contained a letter described the purpose of the study, questionnaire surveys, and a recycling envelope (43). The samples came from Hubei, Hunan, Sichuan, Guangxi, Guangdong, Jiangsu, and Fujian provinces. The data collection lasted for 3 months (April to July 2018).

### Development of Measurement Instrument

Data was collected through a structured self-administered questionnaire. To ensure the validity of the scale, measurement items were adapted from the previous literature. Two experts in Information Management of Wuhan University were invited to review the measurement items before the beginning of the survey. The experts in the IS field were invited to assess logical consistencies, ease of understanding, sequence of items, and contextual relevance. Some minor modifications of the questionnaire were conducted according to their comments. Furthermore, an online pilot test was conducted involving users who had used mobile or computer to seek and share health information on online health community. Based on their comments and suggestions, the measurement items were modified slightly. All survey items were measured using a 5-point Likert scale ranging from 1 (strongly disagree) to 5 (strongly agree).

*Personality service* was measured using the items adapted from Xu et al. (15). The items measured three essential aspects of personality service in online health community, namely, push health information based on online health information behavior, push health information based on interests and hobbies, and push health information based on personal health. *Service quality* was measured using the items adapted from Rodriguez and Edwards (44). These items measured three characteristics of service quality: provide service timely and quickly, service accuracy, and easy to understand. *Health privacy attention* was assessed using 4 items adapted from (45) and (46), the items measured four essential aspects of user health privacy attention, i.e., noticing, encoding, explanting and focusing. *Information sensitivity* was measured using the items adapted from (32). The items measured five essential aspects of user information sensitivity, i.e., information of domestic violence, genetic information, disease information, drug information, and sexual health information. *Social rewards* was assessed using five items adapted from Mohamed and Ahmad (47). These items measured five essential aspects of social rewards in online, that is, expanding social network, getting familiar with new people, helping diagnose and cure diseases, controlling my health status and condition, and getting more treatments. *Health information privacy concerns* was assessed using five items adapted from Malhotra et al. (48). The measured five essential aspects of health information privacy concerns in the online health community include that health information will be stolen by hackers, health information will be resold by websites, health information will be used for conduct propaganda and sales promotion, health information will be used by websites to gain illegal profits, and websites collect too much information about my health. *Trust* was measured using four items adapted from Kankanhalli et al. (19). The measurement focused on trust in doctors and trust in websites. *Reciprocity norm* was measured using four items adapted from Kankanhalli et al. (19) and Chiu et al. (37). The items measured three essential aspects of reciprocity norm in the online health community: in exchange for payment, for help, and for medical support. *Health privacy information self-disclosure* was assessed using four items adapted from Chiu et al. (37) and Kankanhalli et al. (19). The items measured four essential aspects of health privacy information self-disclosure in the online health community, that is, providing health privacy information according to doctors' requirements, active providing health privacy information, frequent providing health privacy information, and planning to provide health privacy information in the future. Table 1 shows all the measurement items involved in this study.

**Table 1:** Constructs and measures.

**Variable**	**Items**	**Sources**
Service quality	SQ1: I can enjoy the services provided by the website in time and quickly.	(44)
	SQ2: The diagnosis service, medication advice, medical knowledge, and popular science information provided by the website are not difficult to understand.	
	SQ3: The diagnosis service, medication advice, medical knowledge, and popular science information provided by the website are scientific and accurate.	
Personality service	PS1: The website will push medical knowledge and popular science information suitable for me according to my behavior on the website.	(15)
	PS2: The website will push the medical knowledge and popular science information I need according to my health status.	
	PS3: The website will push my favorite medical knowledge and popular science information according to my preferences and interests.	
Information sensitivity	IS1: It's sensitive for me to provide information about domestic violence to websites or doctors.	(32)
	IS2: It's sensitive for me to provide genetic information to websites or doctors.	
	IS3: It's sensitive for me to provide mental health information about mental illness, mental illness, etc. to websites or doctors.	
	IS4: It's sensitive for me to provide information to websites or doctors about drug dependence such as sleeping pills and drug abuse such as antibiotics.	
	IS5: It's sensitive for me to provide reproductive / sexual health information about sexual life, sexual orientation, sexually transmitted diseases, adoption, abortion, infertility, etc. to websites or doctors.	
Social rewards	SR1: Health sites help me expand my social network	(47)
	SR2: can get familiar with new people through the health website.	
	SR3: Health websites can help me diagnose and cure diseases.	
	SR4: Health websites can help me establish health records, record my health information, which can monitor and control my health status and condition.	
	SR5: Health websites can help me get more treatments.	
Privacy concerns	PC1: I'm worried that my health information will be stolen by hackers.	(48)
	PC2: I'm worried that my health information will be resold by websites.	
	PC3: I'm worried that my health information will be used for propaganda and sales promotion.	
	PC4: I'm worried that my health information will be used by websites to gain illegal profits.	
	PC5: I'm worried that medical websites collect too much information about my health.	
Reciprocity norm	RN1: If I disclose the necessary personal health information to health website, I will be served accordingly.	(19, 37)
	RN2: If I need help, the website and the doctor will help me.	
	RN3: If health website set up a health file to record my health information, my health status and condition will be monitored and controlled.	
Trust	TR1: I believe that the website will not use my health information for publicity, marketing, resale and other commercial purposes without my permi	(19)
	TR2: I believe that the website will use my health information properly.	
	TR3: I believe that the website doctor will not use my health information for other purposes except medical treatment without my permission.	
	TR4: I believe that the web doctor will do everything possible to solve my problem.	
Self-disclosure intention	SI1: I will disclose my information when the website or my doctor asks me to provide personal health inform.	(19, 37)
	SI2: I plan to use the health website more frequently to obtain personalized services based on my health information.	
	SI3: I plan to provide more personal health information to the website doctors when I am ill.	
	SI4: If my information disclosure helps to maintain health, I will provide my information to any doctor on the website.	
Health privacy attention	HA1: I can select information that has a significant impact on privacy from a large number of health privacy information.	(45, 46)
	HA2: I can quickly analyze the attributes and characteristics of huge health privacy information.	
	HA3: I can quickly categorize a large number of health privacy information.	
	HA4: I can quickly pay attention to the information which related to health privacy from a large number of health information.	

### Samples and Data Collection

Of the 300 questionnaires retrieved in the web-based survey, 264 questionnaires were valid. One hundred fifty-four (58.3%) respondents were female, and 110 (41.7%) were male. The largest age group was 18–28 years old 103 (39.0%), and it was followed by an age group of more than 48 years 109 (41.3%). In terms of the education level, 33.0% (87) of the participants had a bachelor's degree, and 21.6% (57) of the participants had a graduate degree. For health status, participants with common diseases and acute diseases accounted for the largest proportion. One hundred five (39.8%) had common diseases, and 119 (45.1%) had acute diseases. Regarding health checkup frequency, 114 (43.2%) of the participants hardly take health checkup and 136 (51.5%) of them take health checkup once a year. Concerning Internet experience, the largest age group has above 3 years 103 (39.0%) Internet experience, and 15.7% of the rest have 3 years and <3 years Internet experience. Table 2 shows the characteristics of the respondents in this study.

**Table 2:** Demographic statistics.

**Variable**	**Levels**	**Frequency**	**Percentage (%)**
Gender	Male	110	41.7
	Female	154	58.3
Age	<18	25	9.5
	18~28	103	39.0
	31~48	27	10.2
	48~50	86	32.6
	51~60	20	7.6
	≥61	3	1.1
Education	Junior middle school or below	22	8.3
	Senior middle school	56	21.2
	Two year college	42	15.9
	Bachelor	87	33.0
	Master or above	57	21.6
Illness experience	Suffering from common diseases	105	39.8
	Suffering from an acute disease	119	45.1
	Suffering from chronic diseases	5	1.9
	Healthy	12	4.5
	Sub-health	23	8.7
Physical examination frequency	Hardly	114	43.2
	Once a year	136	51.5
	Twice a year	12	4.5
	More than once a year	2	0.8
Internet usage experience	≤1 year	29	11.0
	2 years	27	10.2
	3 years	12	4.5
	≥3 years	196	74.2
Permanent residence	First-tier cities	18	6.8
	Second-tier cities	85	32.2
	Ordinary town	153	58.0
	Countryside	8	3.0

### Data Assessment

The *common method variance* (CMV) issue could be raised through the self-report survey method. This study adopted two methods to assess CMV: Harman's single-factor test and confirmatory factor analysis. We first conducted Harman's single-factor test (49). The largest variance accounted for 20.2% of the total variance, so none of the factors accounted for the majority of the variance. We also performed confirmatory factor analysis (CFA) by loading all the measurement items to only one factor. The results showed a poor fitness, where the chi-square statistic (*x*^2^) = 3,441.691, degrees of freedom (*df*) = 464, *x*^2^/*df* = 7.417 (>3), comparative fit index (*CFI*) = 0.551 (<0.90), normalized fit index (*NFI*) = 0.517(<0.90), incremental fit index (*IFI*) = 0.553 (<0.90), and root mean square error of approximation (*RMSEA*) = 0.156 (>0.08). The test results of both methods imply that CMV is an insignificant threat in our research.

We assessed the nonresponse bias by comparing the questionnaires received in the late (i.e., last 7 days) and early (i.e., first 7 days) stages. The significance levels of all constructs are higher than 0.05, which suggests that the non-response bias is insignificant in this study. We conducted Kruskal–Wallis and Mann–Whitney tests to examine any difference between the online and offline responses (50). The results on the demographic characteristics indicate that the significance levels of age, gender, education, health checkup frequency, Internet experience, and health status are *p* = 0.561, 0.656, 0.488, 0.359, 0.358, and 0.391, respectively, which suggest insignificant differences. Thus, our sample is free from method bias, and the data can be merged for analysis.

### Measurement Model Assessment

We performed a CFA with AMOS 20.0 to evaluate the measurement validity and reliability. The CFA outcomes revealed that the data fit well with the model (*x*^2^ = 1,628.474, *df* = 484, *x*^2^/*df* = 3.365, RMSEA = 0.095, RMR = 0.202, NFI = 0.806, CFI = 0.854, IFI = 0.855). The validity of the constructs includes convergent validity and discriminate validity. We conducted several tests to evaluate convergent validity. First, we evaluated item reliability by calculating the standardized loadings of each item on the construct. Table 3 indicates that each item-to-construct loading was larger than the criterion of 0.70 (51). Therefore, all the measures were sufficiently reliable. Second, we computed Cronbach's alpha and the composite reliability (CR) of each item. Table 4 shows that the minimum CR and Cronbach's alpha values are 0.96 and 0.85, respectively. Both CR and Cronbach's alpha exceeded 0.70, indicating that the instrument is internally consistent and reliable (52). Finally, we examined the average variance extracted (AVE) values (Table 4). All AVE values exceeded the criterion of 0.74, confirming a suitable convergent validity of the measurement model.

**Table 3:** Loadings and cross-loadings.

	SR	RN	TR	IS	PC	SQ	PS	SI	HA
SR1	**0.901**	0.170	0.195	0.109	0.007	0.055	0.086	0.227	0.112
SR2	**0.891**	0.165	0.039	0.109	0.048	0.042	0.102	0.154	0.251
SR3	**0.902**	0.129	0.028	0.054	0.064	0.055	0.112	0.153	0.006
SR4	**0.928**	0.123	0.075	0.101	0.148	0.017	0.157	0.369	0.128
SR5	**0.943**	0.042	0.066	0.022	0.256	0.010	0.116	0.290	0.176
RN1	0.105	**0.821**	0.259	0.079	0.051	0.174	0.234	0.001	0.284
RN2	0.117	**0.909**	0.312	0.041	0.075	0.065	0.269	0.044	0.475
RN3	0.169	**0.880**	0.210	0.001	0.084	0.164	0.196	0.076	0.312
TR1	0.094	0.198	**0.852**	0.107	0.059	0.110	0.093	0.527	0.159
TR2	0.060	0.156	**0.832**	0.093	0.109	0.119	0.173	0.453	0.258
TR3	0.090	0.201	**0.849**	0.099	0.057	0.136	0.058	0.546	0.147
TR4	0.060	0.015	**0.881**	0.156	0.044	0.075	0.142	0.565	0.186
IS1	0.104	0.029	0.076	**0.887**	0.197	0.119	0.163	0.024	0.261
IS2	0.087	0.082	0.003	**0.910**	0.166	0.107	0.154	0.099	0.312
IS3	0.010	0.096	0.198	**0.932**	0.179	0.135	0.124	0.107	0.224
IS4	0.101	0.064	0.182	**0.868**	0.170	0.159	0.138	0.021	0.221
IS5	0.015	0.080	0.147	**0.894**	0.264	0.080	0.140	0.055	0.156
PC1	0.007	0.135	0.325	0.195	**0.910**	0.172	0.228	0.101	0.217
PC2	0.004	0.141	0.263	0.219	**0.949**	0.101	0.195	0.120	0.221
PC3	0.027	0.109	0.244	0.212	**0.923**	0.079	0.150	0.101	0.153
PC4	0.026	0.113	0.250	0.219	**0.783**	0.052	0.162	0.097	0.142
PC5	0.055	0.071	0.209	0.176	**0.894**	0.016	0.182	0.019	0.126
SQ1	0.088	0.292	0.334	0.182	0.247	**0.871**	0.159	0.248	0.259
SQ2	0.063	0.254	0.288	0.151	0.257	**0.896**	0.157	0.240	0.123
SQ3	0.198	0.197	0.319	0.142	0.093	**0.851**	0.163	0.311	0.111
PS1	0.037	0.235	0.374	0.207	0.358	0.173	**0.921**	0.201	0.226
PS2	0.072	0.146	0.320	0.189	0.158	0.155	**0.881**	0.204	0.234
PS3	0.051	0.286	0.253	0.127	0.237	0.181	**0.916**	0.230	0.201
SI1	0.191	0.243	0.146	0.048	0.128	0.144	0.228	**0.832**	0.103
SI2	0.149	0.192	0.129	0.083	0.104	0.151	0.364	**0.882**	0.081
SI3	0.187	0.218	0.113	0.020	0.074	0.080	0.286	**0.921**	0.094
SI4	0.149	0.186	0.126	0.006	0.107	0.172	0.286	**0.879**	0.083
HA1	0.175	0.064	0.155	0.108	0.211	0.210	0.139	0.181	**0.884**
HA2	0.181	0.136	0.118	0.100	0.183	0.230	0.068	0.145	**0.786**
HA3	0.145	0.059	0.147	0.023	0.186	0.218	0.155	0.156	**0.902**
HA4	0.156	0.063	0.124	0.102	0.187	0.225	0.118	0.073	**0.867**

*Bold is the load of the item on the corresponding factor*.

**Table 4:** Descriptive statistics and inter-correlations of the constructs.

**Variables**	**MEAN**	**S.D**	**C.R**	**AVE**	**a**	**1**	**2**	**3**	**4**	**5**	**6**	**7**	**8**	
1.PC	3.706	0.794	0.961	0.851	0.923	**0.922**								
2.RN	3.208	0.746	0.910	0.773	0.851	0.225	**0.879**							
3.TR	3.006	0.784	0.921	0.740	0.881	0.054	0.625	**0.860**						
4.SR	2.615	0.892	0.931	0.874	0.861	0.092	0.307	0.354	**0.935**					
5.IS	3.212	0.808	0.961	0.823	0.942	0.152	0.680	0.714	0.290	**0.907**				
6.SQ	3.422	0.706	0.912	0.783	0.851	0.128	0.489	0.531	0.328	0.518	**0.885**			
7.PS	3.556	0.731	0.934	0.831	0.892	0.178	0.438	0.507	0.264	0.462	0.773	**0.912**		
8.SI	3.094	0.770	0.940	0.792	0.913	−0.017	0.469	0.636	0.406	0.566	0.619	0.542	**0.890**	
9.HA	3.254	0.721	0.912	0.813	0.866	0.121	0.465	0.532	0.524	0.412	0.417	0.359	0.256	**0.902**

^*^P < 0.05, ^**^P < 0.01, ^***^P < 0.001. AVE, Average variance extracted; CR, Composite reliability; SQ, Service quality; PS,Personality service; IS, Information sensitivity; SR, Social rewards; PC, Privacy concerns; RN, Reciprocity norm; TR, Trust; SI, Self-disclosure intention; HA, Health privacy attention. Boldfaced diagonal elements are the square roots of AVEs.

Discriminate validity is defined as follows: the correlations of items do not exist or are relatively weak. Table 4 shows that the square roots of AVE for all the constructs on the diagonal were greater than their corresponding correlation coefficients with other constructs, indicating that the model fulfills the requirements of discriminate validity (52).

### Structural Model Assessment

**Figure 2:**
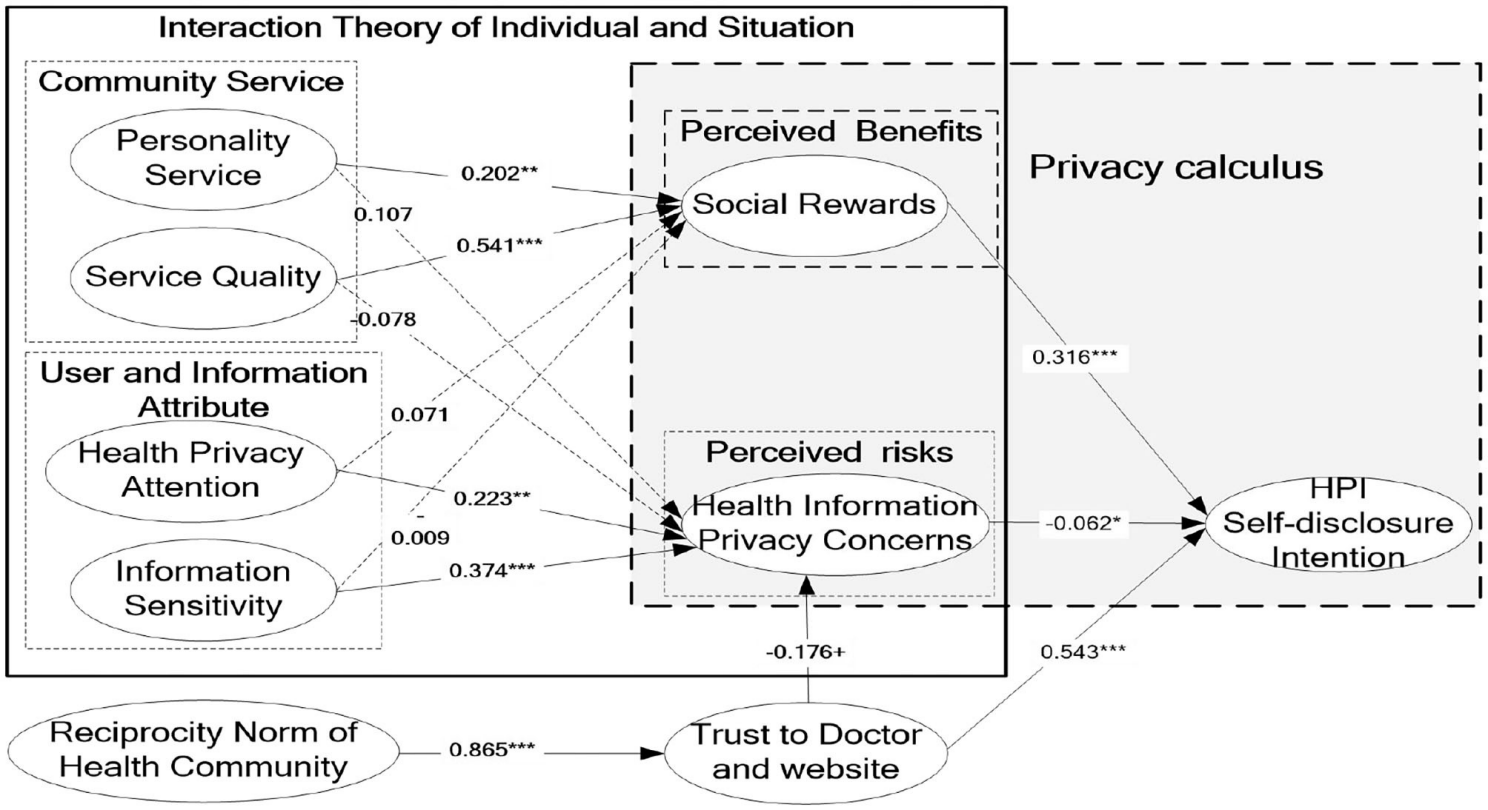
SEM analysis of research model.

A structural model assessment was performed to examine the hypothesized relationships among the constructs. Figure 2 displays the standardized path coefficients and path significances. All the hypothesized relationships were significant with a *p* < 0.05, except H3, H4, H5, and H6. As expected, service quality has a positive effect on the social rewards (β = 0.541, *t* = 4.673, *p* < 0.001) and personality service has a positive effect on social rewards (β = 0.202, *t* = 2.056, *p* < 0.05). Hence, H1 and H2 were supported. Service quality has a insignificant negative effect on the privacy concerns (β = 0.078, *t* = 0.661, *p* > 0.05) and personality service exerted an insignificant positive effect on the privacy concerns (β = 0.107, *t* = 0.771, *p* > 0.05). Thus, H3 and H4 were not supported.

Health privacy attention had a significant effect on the privacy concerns (β = 0.223, *t* = 2.315, *p* < 0.05), while it exerted an insignificant effect on the social benefits (β = −0.071, *t* = 0.074, *p* > 0.05). Thus, H5 was not supported, and H7 was supported. Information sensitivity has a significant impact on privacy concerns (β = 0.374, *t* = 5.673, *p* < 0.001) and exerted an insignificant negative effect on social benefits (β = −0.009, *t* = 0.871, *p* > 0.05). Thus, H5 was not supported and H7 was supported. Social rewards have a significant positive impact on self-disclosure intention (β = 0.316, *t* = 2.754, *p* < 0.05, respectively), and privacy concerns have a significant negative impact on self-disclosure intention (β = −0.062, *t* = 2.054, *p* < 0.05, respectively). H12 and H13 were supported. Finally, reciprocity norm of the health community has significant positive effects on trust (β = 0.865, *t* = 5.232, *p* < 0.001, respectively). Trust had negative impact on privacy concerns (β = −0.176, *t* = 2.232, *p* < 0.01, respectively) and had positive impact on self-disclosure intention (β = 0.543, *t* = 6.232, *p* < 0.001, respectively). Thus, H9, H10, and H11 were supported.

## Discussion

### Major Findings

This study attempts to understand privacy calculus model, reciprocity norms, and web services in online medical communities. Some key findings come from this study. First, this shows that the trade-off will not occur when trust is sufficient to offset the privacy concerns caused by personalized services, reciprocity norms, and other factors. This is different from previous studies. In previous research, privacy trade-offs were the main research direction. Researchers have paid attention to the impact of perceived benefits and perceived risks in e-commerce and social network contexts on privacy self-disclosure, while ignoring that trust is an antecedent variable of privacy self-disclosure (6, 17). Second, for reciprocity norms, this study proposes and verifies a significant positive relationship between reciprocity norms and trust. This shows that the reciprocal norms established by online medical platforms are not always positive. Under the constraint of the equivalent exchange rules, users are forced to share personal health information in order to exchange the medical information on the website, which will improve their concern about health privacy; however, the rewarding personal information contribution behavior generated by gratitude to the website will strengthen users' trust in the website. This is different from previous studies that have always emphasized the positive role of reciprocity norms (19). Third, regarding website services, personality services have a positive impact on both social rewards and privacy concerns, which is consistent with previous research conclusions (15). In particular, the results show that service quality can not only increase the social rewards of users but also reduce the privacy concerns of users. Even though the negative effect of service quality on privacy concerns is not significant, the negative effect may still exist. Fourth, from the perspective of user attributes and website attributes, we also find that information sensitivity and health privacy attention have a positive impact on privacy concerns. This shows that patients' personal information sensitivity and their awareness of personal health privacy are too high, and this will increase users' privacy concerns about the disclosure of personal health privacy information. However, previous studies have not confirmed the relationship among the three items.

### Theoretical Implications

This study has four main contributions. First of all, this study helps us to better understand the important premise of privacy calculus by considering the impact of user's trust. Previous studies analyzed the influencing factors of self-disclosure intention only based on the trade-off between perceived risk and perceived benefit in privacy calculus model (17). However, this theoretical interpretation model causes the trade-off theory to lose the existence basis. Therefore, considering the main effect of independent variables, we propose to consider that trust may be able to completely offset the perceived risk. Once the user does not have the perceived risk, the trade-off will not occur. Our results also confirm this conclusion. The theory of this study shows that there is no privacy trade-off, and self-disclosure intention can be directly formed when trust is sufficient. This conclusion reveals the prerequisite of privacy calculus and the core mechanism of self-disclosure, which make a contribution to privacy calculus theory.

Second, this study verifies differences in the role of reciprocity norms in different scenarios. This paper proposes and empirically tests the change of exchange results caused by scenario change. Most previous studies of social exchange are based on the community in which users voluntarily participate and focus on the positive impact of reciprocity norms (53). For example, Burt's research shows that the knowledge sharing behavior of the Internet community is strongly promoted by reciprocal norms (18), but the negative role of reciprocal norms is ignored. In this study, considering the different background of the community, we explicitly propose that the reciprocity based on the forced nature will increase users' perceived risk in the online community based on the exchange of information equivalence, while reciprocity based on the gratitude nature can promote users' trust. The findings suggest that future research should pay more attention to different backgrounds and develop theories based on them.

Third, this study confirmed the impact of web services on privacy calculus, which is rarely involved in previous studies. Even though some studies based on e-commerce may have tested the relationship between personality service and trust (15, 32), few studies have discussed the relationship between personality service and privacy calculus. Especially, the research of Xu et al. has proved that personality service can enhance users' perception of benefits after personal health privacy information disclosure. However, personalized services also cause serious privacy risks because they require a lot of user information. In this case, it is difficult for researchers and practitioners to determine whether to advocate personalized service (15). A research from Sweeney, Soutar, and Johnson has given us some enlightenment. They think that high service quality (functional quality and technical quality) has a significant negative impact on perceived financial and performance risks. High-quality service can narrow the distance between customers and the company, enhance feelings, and reduce psychological risk of customers (22). Our results are consistent with findings of Xu et al. (15). However, it is worth noting that we have also directly confirmed Sweeney's conjecture, which found a breakthrough for the dilemma of personalized services, and helped us to better understand the importance of website services quality in promoting health privacy self-disclosure.

Fourth, we explored the root factor of privacy concerns from the perspective of user attributes and information attributes. First of all, this study introduces privacy attention of strategic management into this question and empirically confirms the positive impact of user health privacy attention on privacy concerns from the perspective of user attributes. On the one hand, this study expands the theoretical application scope of attention-based view from a new perspective (54, 55). On the other hand, this research has found the core elements that determine privacy concerns from the perspective of users so as to developed the privacy calculus theory. However, the previous studies lacked the research on the relationship between individual privacy characters and privacy concerns. Furthermore, from the perspective of information attributes, although a few studies have suggested a relationship between information sensitivity and users' perceive risks (7, 32), it has not been confirmed. This study directly confirmed the positive relationship between information sensitivity and privacy concerns and also found that the information attribute factors would affect privacy concerns. The findings enriched the privacy calculus research.

### Practical Implications

By considering trade-off basis, exchange principle, and website service, this study provides some important enlightenment on the method to increase users' information disclosure behavior in the online health community. First, contrary to the original view of service providers, our research found that while network service providers pursue personalized services to attract users' attention, they must also focus on improving the quality of website services. Although personalized service can help users of online medical platform expand social network and obtain practical medical information, personality service require users to provide the detailed personal information that will directly lead to increase users' privacy risks (56). In fact, in addition to continue to deepen personalized services, network service providers still need to pay attention to the rationality of web page design, response speed and response time of website upload information, convenience of member communication, security and reliability of website information, and other aspects of website service quality in order to retain users for a long time. These contents help users develop the exhibition of social network and obtain more emotional consolation and high-quality medical information from other netizens; more importantly, the reasonable website service can improve users' sensory emotions and pleasant experience directly. These immersion experiences can reduce users' privacy concerns in the process of health privacy self-disclosure and promote positive health information contribution behavior.

Second, Internet service providers should take measures to enhance users' trust in doctors and websites, since trust is the basic condition for users' willingness to disclose health information. We believe that professional medical information is difficult to obtain while sick users have rigid demand for medical information, so information exchange under involuntary conditions occurs. Such exchange will directly increase user perceived privacy risk and reduce users' trust (57). Therefore, the online health community cannot force users to provide personal information to register as members or comment. On the contrary, the website should open relevant medical information to patients so that users can get sufficient medical assistance on the website. When the user keeps positive emotions for the online health website, such as gratitude and touch, are stimulated, the established reciprocal norms based on voluntary conditions will promote users' trust in doctors and the website. The elimination of privacy calculus psychological process is the basis for users to insist on health information contribution behaviors (58).

Third, from the perspective of information attribute, highly infectious diseases such as the novel coronavirus pneumonia, hepatitis B, and tuberculosis are severely discriminated against in the society. On the other hand, patients will be more sensitive to this kind of information if they are unwilling to disclose such information (59, 60). To solve this problem, website operators or doctors should be very familiar with the sensitivity of patients' health information and distinguish insensitive information from highly sensitive information. Without affecting the diagnosis of the disease, website operators or doctors should not excessively require users to provide sensitive health information and minimize the exposure time and frequency of sensitive information on the website. If there is a need, website operators or doctors should do a good job in information protection and psychological counseling of patients. This would enhance the self-disclosure of health privacy. From the perspective of user attributes, different users pay may have different attention capability on health privacy information. This is the reason for the differences in health privacy concerns and health privacy self-disclosure among individuals. Thus, website operators and doctors should find measures to continuously disturb users' health privacy noticing, health privacy encoding, health privacy interpreting and health privacy focusing process. Such as providing more eye-catching health information, personalized services, information security protection function et al. by this way, users' attention can be shifted, their health privacy attention capability can be weaken, and their health privacy self-disclosure would be enhanced.

### Research Limitations and Future Research Prospects

This study got not only some useful results but also some limitations to be improved in the follow-up studies. First of all, most of the measurement tools used in this paper are from foreign-related research. Although the data results of each variable show good reliability and validity of the scale, the measurement will be more accurate if a scale suitable for the Chinese context can be developed. Second, this paper discusses the impact of service quality and personalized service on users' self-health information disclosure intention. In fact, there are many boundary conditions in this process. In addition to considerations at the website level and individual levels, factors related to personality and cultural background also deserve attention. For instance, shy users may have difficulty sharing their health privacy information under normal circumstances. Furthermore, as we all know, Chinese people tend to save face a lot. Users are reluctant to share some unspeakable diseases with others, causing barriers to sharing users' health privacy information. Third, although there is evidence that the scale measurement in this study has good reliability and validity, the cross-sectional correlation study adopted in this study is not as accurate as the follow-up study in causality judgment. In the future, longitudinal research can be tried to get more objective evaluation results.

## Data Availability Statement

The original contributions presented in the study are included in the article/supplementary material, further inquiries can be directed to the corresponding author/s.

## Ethics Statement

The studies involving human participants were reviewed and approved by Hubei University of Arts and Science, China University of Political Science and Law and Wuhan University Committee.

## Author Contributions

WY is the first author and the main researcher of the study, in which 60% of the whole job have been written and calculated by him with collaboration and assistance of ZY who assisted with polishing the manuscript, ZL gathering and analyzing the data gathering. All authors read and approved the manuscript.

## Conflict of Interest

The authors declare that the research was conducted in the absence of any commercial or financial relationships that could be construed as a potential conflict of interest.
